# Protective Effect of Selected Antioxidants on Naproxen Photodegradation in Aqueous Media

**DOI:** 10.3390/antiox8100424

**Published:** 2019-09-23

**Authors:** Kohei Kawabata, Ayaka Takato, Sayaka Oshima, Shiori Akimoto, Masanori Inagaki, Hiroyuki Nishi

**Affiliations:** 1 Faculty of Pharmacy, Yasuda Women’s University, 6-13-1 Yasuhigashi, Asaminami-ku, Hiroshima 731-0153, Japan; 14141226@st.yasuda-u.ac.jp (A.T.); 14141112@st.yasuda-u.ac.jp (S.O.); inagaki@yasuda-u.ac.jp (M.I.); nishi-h@yasuda-u.ac.jp (H.N.); 2 Graduate School of Biomedical and Health Sciences, Hiroshima University, 1-2-3 Kasumi, Minami-ku, Hiroshima 734-8553, Japan; akimotos@hiroshima-u.ac.jp

**Keywords:** naproxen, photodegradation, antioxidant, photoprotective effect, L-ascorbic acid, HPLC

## Abstract

A photostabilization strategy is an important aspect of quality assurance for photosensitive compounds. This study focused on the photoprotective effects of selected antioxidants including the effect of L-ascorbic acid (AA) on naproxen (NX) photodegradation in aqueous media. NX degradation during ultraviolet light (UV) irradiation and the protective effects of selected antioxidants were monitored by high-performance liquid chromatography (HPLC). The addition of AA induced the suppression of NX photodegradation, although the protective effect disappeared after AA was degraded completely. The results of the evaluations on the photoprotective effects on NX photodegradation and antioxidative activities of AA and other antioxidants showed that the protective effects of antioxidants are dependent on reducing power and photostability under UV irradiation. In this experiment, quercetin (QU) is the most effective antioxidant on account of the residual rate of QU after UV irradiation and the antioxidative activity in the potential antioxidant (PAO) test was significantly higher compared to other antioxidants following the higher protective effect on NX photodegradation.

## 1. Introduction

It is well known that exposure to ultraviolet light (UV) irradiation, which is present in sunlight, can induce changes in the properties of chemical compounds. The energy derived from UV irradiation is absorbed by compounds in the ground state following the formation of an excited state that triggers a photochemical reaction, such as bond cleavage and addition of the functional group. The variety of chemical structures contributes to various photochemical reactions and their outputs as photoproducts. Chemical compounds that tend to be affected by UV irradiation are called photosensitive compounds. Photosensitive compounds, including chemical UV filters and pharmaceuticals, are reported to be degraded or converted to products by UV irradiation [1,2,3,4,5]. Photodegradation may provoke the loss of the beneficial effects of these compounds and the gain of adverse effects on account of the generation of toxic photoproducts. It is reported that the biological effects of some pharmaceuticals are changed by UV irradiation [6,7,8] on account of their photoconversion to photoproducts. Various photochemical reactions may be induced by UV irradiation [9,10,11]. These reports indicate that the generation of various photoproducts is dependent on these chemical reactions. The photosensitivity of chemical compounds is a major determinant of their quality and quantity.

To date, there are a lot of reports focused on the photostability and photoconversion of various pharmaceuticals. These pharmaceuticals have been reported as not stable for UV irradiation and undergo conversion processes following the generation of their photoproducts. In the case of nifedipine, an anti-hypertensive drug, UV irradiation generates reactive oxygen species such as singlet oxygen and superoxide species during the photodegradation process [12] as a result of photoproduct determination. Furthermore, additional research showed that the oxidation of the dihydropyridine ring following the generation of pyridine derivatives was the main degradation mechanism [7,13,14]. There are some reports showing the more complex photochemical reaction of nifedipine with the formation of further photoproducts [10,15,16]. Moreover, photoproducts of dihydropyridine have no therapeutic effects [7,8]. From these results, it is indicated that UV irradiation contributes to the loss of the beneficial effects of nifedipine. These reports show that photodegradation affects the behavior of a photosensitive pharmaceutical, and the photostability of the pharmaceutical is an important factor in retaining the therapeutic effects of a pharmaceutical after UV irradiation. 

To overcome the photostability problem of photosensitive pharmaceuticals, photostabilization strategies have been developed in recent years [17]. For example, UV filtering, such as solar filters and encapsulation, is a protective barrier against UV irradiation to protect photosensitive compounds. On the other hand, the addition of an antioxidant is one of the photostabilization strategies derived from the deactivation of the excited state of photosensitive compounds and the reaction with free radicals and reducing reactive oxygen species. There are several reports indicating that the addition of UV filtering and the antioxidant are effective methods for the photostabilization of some pharmaceuticals [18,19,20]. The encapsulation approach is the major method of photostabilization strategies, followed by the addition of antioxidants and solar filters, and cyclodextrins have shown the most promising results for improvement in chemical stability especially [17]. However, in some cases, a stabilization effect provided by encapsulation was decreased [21], which suggests that the use of a photostabilizer is limited and the development of various photostabilization strategies is needed for the photoprotection of various photosensitive compounds. To the best of our knowledge, there are few reports focused on the comparison of the photostabilization effects of various antioxidants for photosensitive pharmaceuticals.

In this study, the protective effects of selected antioxidants on naproxen (NX), a non-steroidal anti-inflammatory drug (NSAID), photodegradation were evaluated. There are several reports indicating that NX is a photosensitive pharmaceutical that can be photodegraded by UV irradiation and sunlight [2,22]. First, the photoprotective effect of L-ascorbic acid (AA) was investigated. Photostabilization by AA for NX photodegradation and its dose dependency were evaluated. Second, the photoprotective effects of selected antioxidants were determined as in AA. Finally, the antioxidative effects of test antioxidants were evaluated by means of a test kit for the Potential Antioxidant (PAO test). The aim of this research is to determine what is essential in a good photostabilizer.

## 2. Materials and Methods 

### 2.1. Material

NX, AA, isoascorbic acid (IA), L-ascorbic acid-2-phosphate trisodium (AA-P), methanol and acetic acid were purchased from Fujifilm Wako Pure Chemical Corporation (Osaka, Japan). Quercetin (QU), catechin (CA) and curcumin (CU) were purchased from Tokyo Chemical Industry Corporation (Tokyo, Japan). L-Ascorbic acid-2-glucoside (AA-G) was obtained from Hayashibara Corporation (Okayama, Japan). Milli-Q water (18.2 mΩ/cm) was prepared by using a Milli-Q water purification system (Millipore, Billerica, Burlington, MA, USA). 

### 2.2. Methods

#### 2.2.1. Preparation of a Test Solution of NX

A test solution of NX (86.9 µmol/L) was prepared as follows: 10 mg of NX was dissolved in 1 mL of methanol, and this solution was diluted further with Milli-Q water to achieve a concentration of 86.9 µmol/L. A volume of 9 mL of diluted solution in a glass vial was used for the UV irradiation experiment.

#### 2.2.2. UV Irradiation Experiment

UV irradiation was carried out in a light cabinet equipped with a 20 W FL20S BLB black light lamp (Toshiba, Tokyo, Japan). The most abundant wavelength of the emission light from this lamp was 365 nm, and its irradiation intensity value was 500 µW/cm^2^/sec. The irradiation intensity was measured using a digital radiometer equipped with a 365 nm sensor (UVX-36, UVP, Upland, CA, USA). Control samples were kept under the same condition but were covered with an aluminum foil to block UV irradiation.

#### 2.2.3. High-Performance Liquid Chromatography (HPLC) Method for the Quantitative Determination of NX and Antioxidants

A volume of 20 µL of irradiated sample solutions were injected into a Prominence high-performance liquid chromatography (HPLC) system, which is composed of a LC-20 AB pump, a SIL-20 AC autosampler, a SPD-M20A photodiode array detector with LCsolution software, a CBM-20A system controller, a DGU-20A3 degasser, and a CTO-20A column oven (Shimadzu Corporation, Kyoto, Japan). The analytical column was a Shim-pack VP-ODS column (5 µm, 4.6 × 150 mm, Shimadzu Corporation, Kyoto, Japan). The column was kept at 40 °C. Isocratic separation was achieved using a mobile phase composed of methanol and acetic acid (50% methanol containing 0.1% acetic acid, v/v), which was maintained at a flow rate of 1.0 mL/min. Amounts of NX and test antioxidants evaluated by HPLC were shown as the residual rate for amounts before UV irradiation.

#### 2.2.4. Evaluation of Selected Antioxidant Photostabilization for NX

Selected antioxidants (AA, IA, AA-P, AA-G, QU, CA and CU) were dissolved in 50% methanol to achieve a concentration of 10 mmol/L. In the photostabilization experiment, a test solution (9 mL) was prepared using a methanol solution of NX (43.4 mmol/L) and Milli-Q water to achieve a concentration of 86.9 µmol/L of NX and 100 µmol/L of antioxidants. Thereafter, the black light lamp irradiated these test solutions. The irradiation time was up to 3 h at 20 °C. Control samples were kept under the same condition in the absence of these antioxidants. Residual amounts of NX and selected antioxidants were determined by means of HPLC in the same condition. Also, AA solutions of 1 mmol/L, 100 mmol/L and 1 mol/L were prepared to evaluate the dose dependency of AA. These AA solutions were added to the NX solution to achieve a concentration of AA of 10 µmol/L, 1 mmol/L and 10 mmol/L, and the black light lamp irradiated these test solutions. For the evaluation of the time dependency of the protective effect of AA (1 mmol/L), the irradiation time was up to 12 h in the absence and in the presence of AA at 20 °C. All experiments were carried out in triplicate. Kinetics constants of NX degradation and selected antioxidants were determined by means of the calculation of the slope of approximation straight line using Microsoft Excel.

#### 2.2.5. Evaluation of Antioxidative Potencies

The antioxidative potencies of testing antioxidants were evaluated using the PAO test (Nikken SEIL Corporation, Shizuoka, Japan). This assay evaluated the Cu^+^ level derived from the reduction of Cu^2+^ induced by the antioxidative potency by means of the spectrophotometer. From these results, the antioxidant capacity of the sample was calculated as copper-reducing power (µmol/L). The antioxidants were dissolved in 50% methanol to achieve a concentration at 100 µmol/L for the PAO test. All experiments were carried out in triplicate.

### 2.3. Statistical Analysis

Data are expressed as the mean ± standard deviation (S.D.). The homogeneity of variance was established using a one-way ANOVA. Statistical significance between two groups was estimated by the Student’s *t*-test, and between more than three groups was estimated by Tukey’s test. The threshold for assessing significance was *p* < 0.05 or *p* < 0.01.

## 3. Results and Discussion

### 3.1. Photodegradation Kinetics of NX and the Protective Effect of AA

**Figure 1:**
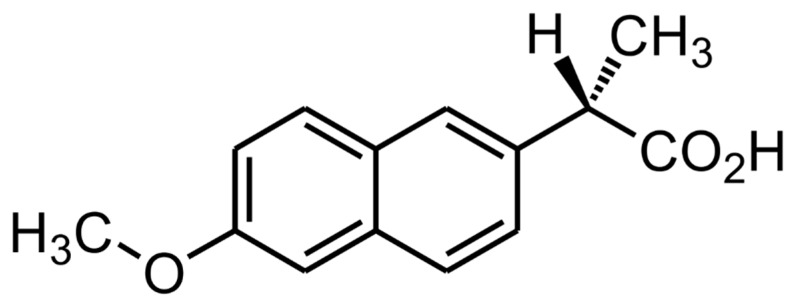
Chemical structure of naproxen (NX).

The photosensitivity of chemical compounds is an important factor for the determination of their nature, which may be influenced by UV irradiation. Therefore, photostabilization strategies, including the addition of antioxidants, are necessary for safe use. In this study, the photoprotective effects of selected antioxidants for NX photodegradation were examined and their effectiveness as photostabilizers was evaluated. The chemical structure of NX is shown in Figure 1. NX is well known as a photosensitive pharmaceutical, and some reports indicate the photodegradable property of NX induced by UV irradiation and sunlight irradiation [2,22].

**Figure 2:**
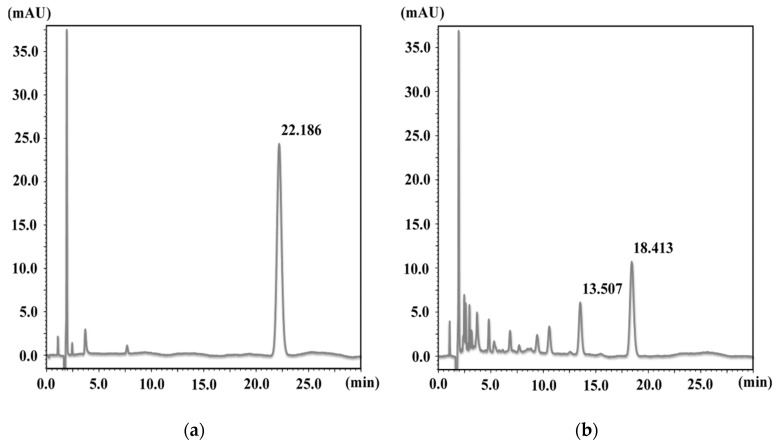
HPLC chromatogram of the (**a**) NX solution and the (**b**) NX solution after UV irradiation for 3 h.

At first, the kinetics of NX photodegradation in aqueous media and the photoprotective effect of AA, which is one of major antioxidants, were examined. HPLC chromatograms of NX and UV-irradiated NX for 3 h are as shown in Figure 2. 

**Figure 3:**
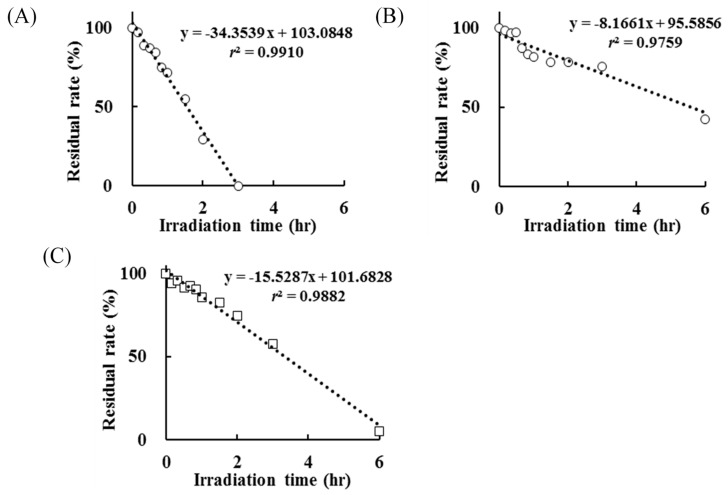
Residual rate of (**A**) NX in the absence of L-ascorbic acid (AA), (**B**) NX in the presence of AA at 1 mmol/L and (**C**) AA after UV irradiation for up to 6 h.

The retention time of NX was 22.2 min and this peak disappeared after UV irradiation for 3 h, with the generation of peaks of various photoproducts. The results of NX photodegradation in the absence or presence of AA showed that the degradation was decreased by an addition of 1 mmol/L AA (Figure 3A,B). 

In the presence of AA, NX persisted after UV irradiation for 3 h and its residual rate was 75.43%. Additional irradiation, as a result of UV irradiation for up to 12 h, induced the complete NX degradation. The kinetics constants of NX photodegradation in the absence and presence of AA were 34.35 and 8.17, respectively, which suggested that AA suppressed the degradation rate of NX induced by UV irradiation to less than 25% on account of its antioxidative potency or filter activity (change in kinetics constants on account of AA: 1.00 to 0.24). Also, the photodegradation rate of AA, which was added to the test solution, was monitored by means of HPLC and it was almost completely degraded after UV irradiation for 6 h (Figure 3C). The antioxidative property of AA is derived from the release of hydrogen from the 2,3-enediol moiety following conversion to dehydroascorbic acid, which has no antioxidative potency, and this reaction proceeded immediately at room temperature by the photoirradiation [23]. These findings show that an AA solution is unstable due to oxidation by the dissolved oxygen on UV irradiation. In this experiment, the control sample was not influenced for 12 h. The residual rates of NX and AA in the control sample after UV irradiation for 12 h were 101.22% and 99.87%, respectively. It is indicated NX and AA degradation were not due to other factors such as hydrolysis and temperature, and only UV irradiation contributed to NX and AA degradation.

### 3.2. Time Dependency and Dose Dependency of the Photoprotective Effect of AA

**Figure 4:**
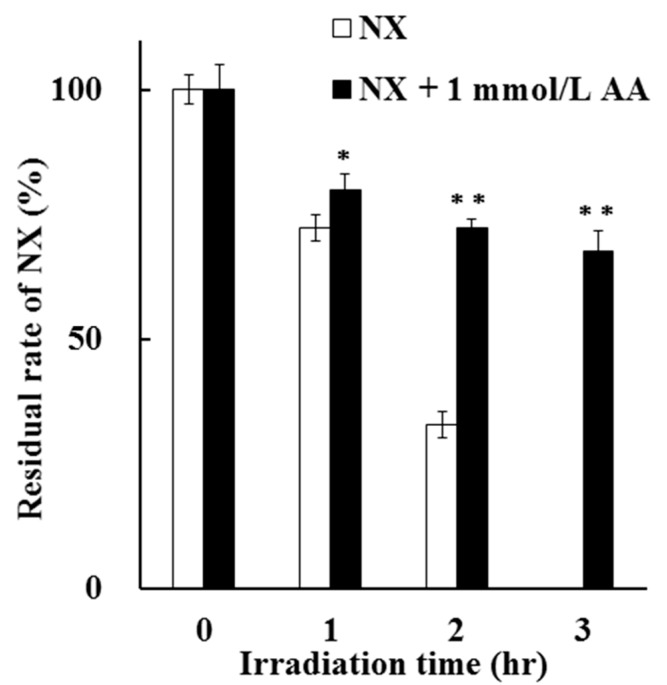
Photoprotective effect of 1 mmol/L AA on NX photodegradation in a solution for up to 3 h. The values represent the mean ± S.D. (*n* = 3). * Difference compared with NX (*p* < 0.05). ** Difference compared with NX (*p* < 0.01).

A comparison of the residual rate of NX in a solution after UV irradiation up to 3 h in the absence and presence of 1 mmol/L AA is shown in Figure 4. The residual rate of NX in the presence of AA was significantly higher than that in the absence of AA after UV irradiation for 1 to 3 h. After UV irradiation for 3 h, the residual rate of NX in the presence of AA was 67.79%, which is higher compared to that in the absence of AA. These results indicate the significant photostabilization effect of AA on NX photodegradation in a solution.

**Figure 5:**
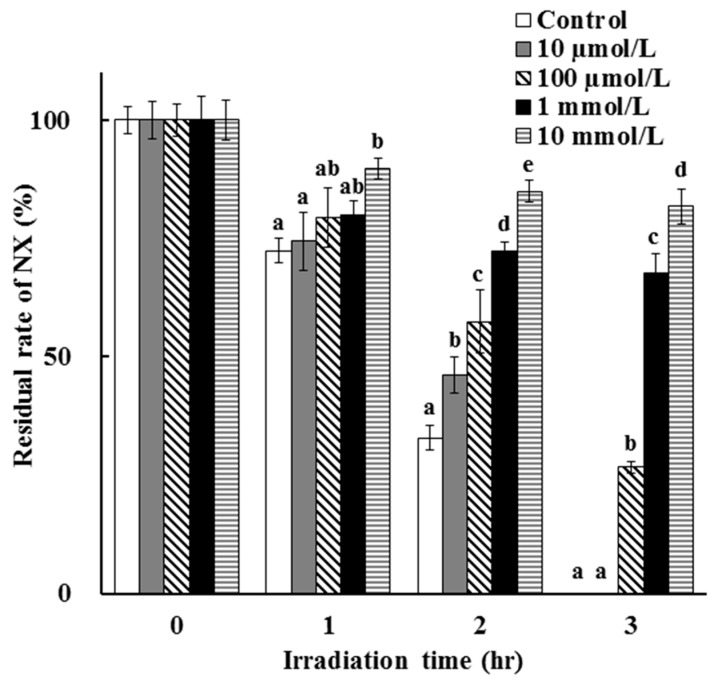
Dose dependency of the photoprotective effect of AA on NX photodegradation in a solution. The values represent the mean ± S.D. (*n* = 3). ^a–e^ Means without a common superscript are significantly different (*p* < 0.05).

The dose dependency of the photostabilization effect of AA was evaluated and the result is as shown in Figure 5. It is suggested that NX was photodegraded slower in the presence of AA at a higher concentration. Also, AA at a lower concentration tends to be degradable and shows a small photoprotective effect on NX degradation. This is as expected, since the antioxidative properties and filtering activity of AA are higher at higher concentrations in the test solution after UV irradiation. After the disappearance of AA due to its degradation, the photostabilization effect was lost and NX photodegradation proceeded with no barrier.

The photostabilization effect of AA in this study is in agreement with previously reported findings [20,24,25]. It is reported that diclofenac, a photosensitive pharmaceutical, in semisolid formulations containing 2.5–5% AA showed high stability under UV irradiation [20]. Furthermore, dithanol photodegradation was inhibited by the combination of AA and other photostabilization strategies such as nanocapsules [26]. It is indicated that the antioxidative activity of AA contributes to the photoprotective effect. As in other reports, the disadvantage of AA, from the perspective of photostabilization, is that it shows a loss of photoprotective effect since it is photodegradable. It is suggested that more photostability and antioxidative activity are necessary for improvement in the photostabilization strategy using antioxidants.

### 3.3. Comparison of the Photoprotective Effect of Selected Antioxidants

**Figure 6:**
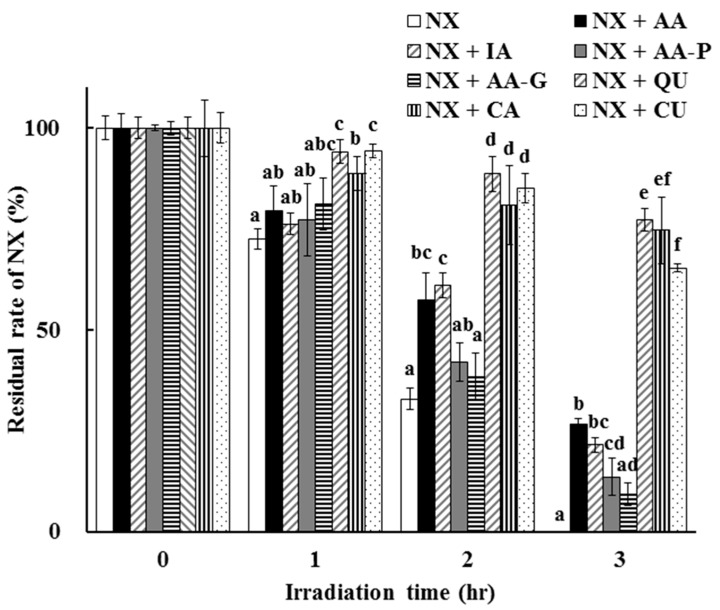
Photoprotective effect of 100 µmol/L selected antioxidants on NX photodegradation in a solution. The values represent the mean ± S.D. (*n* = 3). ^a–f^ Means without a common superscript are significantly different (*p* < 0.05). Isoascorbic acid (IA), L-ascorbic acid-2-phosphate trisodium (AA-P), L-Ascorbic acid-2-glucoside (AA-G), quercetin (QU), catechin (CA) and curcumin (CU).

The photoprotective effects of other antioxidants, including IA, AA-P, AA-G, QU, CA and CU, on NX photodegradation were determined and compared to AA. A comparison of the residual rate of NX after UV irradiation up to 3 h in the presence of each antioxidant at a concentration of 100 µmol/L is shown in Figure 6. 

NX photodegradation in a solution was induced by UV irradiation but the degradation rate was significantly different among seven antioxidants. In the case of IA, which is a stereoisomer of AA, the residual rate of NX after UV irradiation for 2 to 3 h was almost the same as in the case of AA. The residual rates of NX in the presence of AA and IA were 60.90 ± 3.95% and 63.66 ± 3.50% after UV irradiation for 2 h, and 26.65 ± 1.25% and 21.44 ± 1.86% for 3 h, respectively. However, AA-P and AA-G, which are AA derivatives, showed a lower photostabilization effect compared to AA and IA, and the residual rates of NX in the presence of AA-P and AA-G after UV irradiation for 3 h were 13.56 ± 4.53% and 9.28 ± 2.83%, respectively. AA-P and AA-G persisted after UV irradiation for 3 h (residual rate: 49.65 ± 5.18% and 53.99 ± 5.11%, respectively) although AA and IA were degraded completely (Table 1). The antioxidative activities of AA, IA, AA-P and AA-G evaluated by the PAO test indicated that AA-P and AA-G showed a reducing power significantly less than AA and IA. From these results, it is tempting to speculate that AA-P and AA-G are resistant to UV irradiation due to the inhibition of their own oxidation. However, in these antioxidants, weakened hydrogen release from the 2,3-enediol moiety induced a decrease in antioxidative activity in exchange for the suppression of the conversion to dehydroascorbic acid. AA-P and AA-G are the AA derivatives that are *O*-substituted at the C-2 position of AA and designed for improvement in the instability of AA. Some reports showed their utility for the purpose of the radical scavenging [27,28], but the protective effect of AA-P and AA-G on NX photodegradation was less than AA in this study.

On the other hand, other antioxidants, including QU, CA and CU, showed a greater photoprotective effect on NX photodegradation in a solution than AA, IA and AA derivatives. The residual rates of NX in the presence of QU, CA and CU after UV irradiation for 2 to 3 h were significantly higher than those in the presence of AA, IA, AA-P and AA-G (Figure 6). The residual rates of NX in the presence of QU, CA and CU after UV irradiation for 2 h were 88.66 ± 4.31%, 80.99 ± 9.78% and 85.17 ± 3.61%, and 77.24 ± 2.84%, 74.62 ± 8.18% and 65.34 ± 1.10% for 3 h, respectively. As a result of the PAO test, the antioxidative activities of QU, CA and CU were significantly higher than that of AA, IA and AA derivatives. The values of the reducing power (µmol/L) were as follows: QU, 1614.95 ± 229.75, CA, 1195.92 ± 72.18, CU, 899.59 ± 94.30, respectively (Table 1). Furthermore, the residual rates of QU and CA after UV irradiation for 3 h were 78.71 ± 9.08% and 62.45 ± 3.53%, respectively, which showed that QU and CA were more resistant to UV irradiation in addition to having greater antioxidative potencies compared to the other antioxidants. These results suggested that QU and CA might act as more efficient photostabilizers for NX photodegradation. Both QU and CA have a polyphenol backbone, which is well known in antioxidants, and there are some reports focused on their antioxidative behavior [25,29]. Furthermore, residual rates of over 50% after UV irradiation at 312 nm for 6 h have been reported for flavonoids including CA [30]. In the case of CU, however, the residual rate of NX in the presence of CU after UV irradiation for 2 to 3 h and the reducing power were comparable to those in QU and CA, although CU was completely degraded by UV irradiation (Table 1, Figure 6). CU is also a polyphenol compound and several studies mention observed antioxidant activity [31,32]. Furthermore, it is reported that CU is unstable under UV irradiation and is photo-converted to several photoproducts and two of them are ferulic acid and vanillic acid [33], which have antioxidative potency. From these results, it is postulated that photoproducts of CU generated by UV irradiation might have protective effects on NX photodegradation following higher persistence after UV irradiation, although CU was degraded completely. Photoproducts of CU were not detected in the HPLC analysis in this study. From these results and other reports focused on CU photoproducts, the photoprotective effect of CU might be derived from the generation of its photoproducts in addition to antioxidative activity.

## 4. Conclusions

This work revealed that both the antioxidative potency and the photostability of antioxidants contribute to photostabilization effects on the photodegradation of the studied pharmaceutical. The addition of selected antioxidants, including QU, CA and CU, to photosensitive pharmaceuticals such as NX might be a good strategy for improvement in stability under UV irradiation. Increasing photostability is important for the formulation of medicine, especially medicines with photodegradable potency. Further research, including the evaluation of other chemical compounds, is required for the development of a photostabilization strategy.

## Figures and Tables

**Table 1:** Photoprotective effects and antioxidative activities of selected antioxidants.

	Residual Rate of NX after UV Irradiation for 3 h	Residual Rate of Antioxidants after UV Irradiation for 3 h	Kinetics Constant of NX Photodegradation	Antioxidative Activity
	(%)	(%)	(h^−1^)	(µmol/L)
Control	0.00 ± 0.00 ^a^	−	33.96	−
AA	26.65 ± 1.25 ^b^	0.00 ± 0.00 ^a^	24.21	515.28 ± 24.06 ^a^
IA	21.44 ± 1.86 ^bc^	0.00 ± 0.00 ^a^	25.08	536.12 ± 30.27 ^a^
AA-P	13.56 ± 4.53 ^c,d^	49.65 ± 5.18 ^b^	29.45	121.72 ± 17.48 ^b^
AA-G	9.28 ± 2.83 ^a,d^	53.99 ± 5.11 ^b^	31.48	54.58 ± 4.01 ^b^
QU	77.24 ± 2.84 ^e^	78.71 ± 9.08 ^c^	7.38	1614.95 ± 229.75 ^c^
CA	74.62 ± 8.18 ^e,f^	62.45 ± 3.53 ^b^	8.39	1195.92 ± 72.18 ^d^
CU	65.34 ± 1.10 ^f^	0.00 ± 0.00 ^a^	11.31	899.59 ± 94.30 ^e^

The values represent the mean ± S.D. (*n* = 3). Means in the same row without a common superscript (^a–f^) are significantly different (*p* < 0.05).
